# Supplementary material for: Lifestyle Pathways Affecting Children’s Mental Health in Japan during the COVID-19 Pandemic
Source: Children (Basel). 2023 May 26;10(6):943. doi: 10.3390/children10060943 (PMC10297420; doi:10.3390/children10060943)
Supplement: Supplementary file 1 [file children-10-00943-s001.zip › children-2355888-supplementary.pdf]

Table S1. Factor structure for pre-school children.

Variable	*M*	*SD*	I	II	III	IV	V
I "Sleeping habits" (α=.78)							
1. bedtime on the holidays	2.79	.72	.83	.01	.03	-.03	.01
2. wake-up time on the holidays	1.87	.71	.62	.01	-.03	-.01	.01
3. wake-up time on the weekdays	2.51	.61	.52	-.02	-.01	.05	-.05
4. bedtime on the weekdays	2.98	.64	.77	-.01	.01	.03	.01
II "Physical/psychosocial health (PPH)" (α=.71)							
5. Do you feel tired as soon as you do some-thing?	3.20	.86	-.03	.58	.09	-.04	.03
6. Do you yawn from the morning?	3.13	.89	.11	.54	-.04	.02	-.01
7. Do you have abdominal pain?	3.36	.77	-.01	.54	-.01	-.16	.02
8. Do you feel sleepy in the daytime?	2.99	.91	-.01	.49	-.04	.04	.00
9. Do you feel sick?	3.73	.55	.00	.51	.00	-.08	.00
10. Do you feel irritated?	2.68	.91	.01	.50	.00	.16	-.06
11. Do you wake up in the middle of your sleep?	3.07	.90	-.08	.43	-.01	.09	-.01
III "Physical activity" (α=.76)							
12. Do you use a variety of exercise and playground equipment?	3.16	.77	.00	-.02	.78	.04	-.05
13. Do you exercise to the point of sweating?	3.11	.78	.00	-.02	.73	-.02	-.01
14. Do you play outside?	3.09	.72	.00	.04	.60	-.02	.02
15. Do parents and children engage in physical exercise together?	2.70	.77	.00	.00	.53	-.01	.09
IV "Life skills" (α=.74)							
16. Can you fold your own clothes when you take them off?	2.83	.95	-.04	-.04	.04	.75	-.06
17. Can you clean up after playing?	2.73	.81	-.02	.02	.03	.64	-.03
18. Do you try to get ready for the next day by yourself?	2.58	.98	.08	-.04	-.05	.59	.03
19. Can you eat in a well manner?	2.74	.74	.01	.05	-.02	.59	.10
V "Healthy diet" (α=.77)							
20. Can you eat without liking it?	2.49	.88	-.02	.01	.00	.07	.90
21. Do you like vegetables?	2.63	.88	.00	-.02	.03	-.05	.69
first eigen value			3.99	2.27	2.07	1.81	1.21
contribution (%)			19.02	29.83	39.68	48.31	54.05

Table S2. Factor structure for elementary school children.

Variable	*M*	*SD*	I	II	III	IV	V
I "Life skills" (α=.78)							
1. Can you clean up after playing?	2.88	.86	.78	-.01	.01	.00	-.06
2. Can you fold your own clothes when you take them off?	2.88	.94	.77	-.04	.00	.05	-.04
3. Can you eat in a well manner?	3.04	.76	.59	.01	-.01	-.04	.12
4. Do you try to get ready for the next day by yourself?	3.13	.90	.58	.05	-.02	.02	.02
II "Physical/psychosocial health (PPH)" (α=.72)							
5. Do you have abdominal pain?	3.08	.92	-.09	.61	-.05	-.02	.03
6. Do you feel sick?	3.57	.69	-.05	.56	-.07	-.05	.06
7. Do you feel tired as soon as you do some-thing?	3.10	.93	.04	.56	.17	-.05	.01
8. Do you feel sleepy in the daytime?	3.19	.91	-.02	.54	-.02	.13	-.05
9. Do you yawn from the morning?	2.77	1.04	.01	.52	.00	.14	-.05
10. Do you feel irritated?	2.45	.96	.15	.45	.01	-.01	-.01
11. Do you wake up in the middle of your sleep?	3.27	.83	.03	.43	-.02	-.13	-.02
III "Physical activity" (α=.78)							
12. Do you use a variety of exercise and playground equipment?	2.93	.92	.04	-.02	.76	.01	-.02
13. Do you exercise to the point of sweating?	3.12	.86	-.04	-.01	.75	-.07	.04
14. Do you play outside?	2.79	.91	-.03	.00	.68	.03	.00
15. Do parents and children engage in physical exercise together?	2.54	.90	.02	.00	.56	.05	.00
IV "Sleeping habits" (α=.75)							
16. bedtime on the holidays	2.31	.78	-.02	.00	.00	.88	.02
17. bedtime on the weekdays	2.66	.70	.00	-.05	.01	.81	.00
18. wake-up time on the holidays	1.89	.79	-.03	.05	.00	.54	.03
19. wake-up time on the weekdays	2.90	.42	.09	-.03	-.01	.40	-.02
V "Healthy diet" (α=.71)							
20. Can you eat without liking it?	2.92	.90	.06	.03	-.03	.01	.81
21. Do you like vegetables?	3.00	.89	-.04	-.03	.04	.02	.67
first eigen value			3.98	2.38	1.98	1.92	1.26
contribution (%)			18.97	30.32	39.76	48.91	54.91
